# Computed Tomography Image under Artificial Intelligence Algorithm to Evaluate the Nursing and Treatment Effect of Pemetrexed Combined Platinum-Based Chemotherapy on Elderly Lung Cancer

**DOI:** 10.1155/2022/2574451

**Published:** 2022-06-06

**Authors:** Qing Gu, Shu'e Li

**Affiliations:** Department of General Medicine, The First Affiliated Hospital of Suzhou University, Suzhou 215006, Jiangsu, China

## Abstract

This study was to evaluate the clinical efficacy of pemetrexed combined with platinum-based chemotherapy in the treatment of elderly lung cancer using electronic computed tomography (CT) images based on artificial intelligence algorithms. In this study, 80 elderly patients with lung cancer treated were selected and randomly divided into two groups: patients treated with pemetrexed combined with cisplatin were included in the pemetrexed group and patients treated with docetaxel combined with cisplatin were included in the docetaxel group, with 40 cases in each group. The DenseNet network was compared with the Let Net-5 and ResNet model and applied to the CT images of 80 elderly patients with lung cancer. The diagnosis accuracy of the DenseNet network (97.4%) was higher than that of the Let Net-5 network (80.1%) and ResNet model (95.5%). Carcinoembryonic antigen (CEA), cytokeratin fragment antigen 21–1 (CYFRA 21–1), and squamous cell-associated antigen (SCC) after chemotherapy in the pemetrexed group and docetaxel group were all lower than those before chemotherapy, showing statistically obvious differences (*P* < 0.05). The satisfaction degree of nursing care in the pemetrexed group (92.67%) was significantly higher than that in the docetaxel group (85.62%), and the difference was statistically significant (*P* < 0.05). Adverse reactions such as fatigue, diarrhea, and neutrophils in the pemetrexed group were lower than those in the docetaxel group, and the difference was statistically great (*P* < 0.05). The DenseNet convolutional neural network has high diagnostic accuracy; methotrexate combined with platinum chemotherapy can improve the chemotherapy effect in elderly patients with lung cancer, with low degree of adverse reactions and good overall tolerance, which can be used as the first-line treatment for elderly patients with lung cancer.

## 1. Introduction

Lung cancer is the malignant tumor with the highest morbidity and mortality in the world, and it is increasing year by year [1, 2]. Nonsquamous non-small cell lung cancer (NSCLC) is a common pathological type of lung cancer. Because of its insidious onset and rapid disease progression, patients are usually in the advanced stage when diagnosed, and 50% of the elderly are over 65 years old, and most of them are in the advanced stage, so they cannot be treated by surgery [3–5]. For a long time, platinum-containing dual-drug chemotherapy has been the first-line standard treatment for patients with advanced NSCLC without driver gene mutations. Pemetrexed combined with platinum chemotherapy has been approved to be the first-line treatment way by the Food and Drug Administration of the United States for the treatment of patients with advanced nonsquamous NSCLC [6]. In China, pemetrexed combined with platinum-based chemotherapy is more widely used in nonsquamous NSCLC patients. At present, the efficacy and safety of pemetrexed combined with platinum in the first-line treatment of nonsquamous NSCLC patients have not been clearly evaluated.

In recent years, with the development of medical image informatics, digging out image features and analyzing clinical information from medical images has gradually attracted the attention of clinicians [7–9]. Studies have confirmed that computed tomography (CT) scans have the advantages of noninvasive and repeated examinations and can evaluate the efficacy of tumors based on tumor size, enhancement characteristics, and density changes [10]. After chemotherapy, the efficacy of chemotherapy can be assessed noninvasively based on the patient's clinical indicators, CT indicators, and histological indicators, and a personalized treatment plan suitable for the patient can be selected, which can provide a good reference for new treatment methods.

The function of automatic search and representation by artificial intelligence is very useful in the medical field. The main features can be extracted by artificial intelligence, and CT images can be classified without human intervention. The newly developed DenseNet is a convolutional neural network (CNN) with dense connection function, in which any two layers are directly connected; the input of each layer is the union of the outputs of all the previous layers, and the characteristic map of this layer is also the input of all the subsequent layers [11]. Wan et al. (2021) [12] found that DenseNet is more efficient than a convolutional neural network, which is mainly reflected in the reduction of computation and the reuse of features in all layers of the network. DenseNet can effectively detect and classify pulmonary nodules in CT images for accurate diagnosis.

In this study, 80 elderly patients with lung cancer were selected, and the artificial intelligence algorithms were innovatively combined with CT images to segment and calibrate the elderly patients with lung cancer so as to explore the efficacy of pemetrexed combined with platinum-based chemotherapy in the treatment of lung cancer and truly realize the desire of “early detection, early diagnosis, early treatment, and early cure.”

## 2. Research Objects and Grouping

### 2.1. Research Objects

In this study, 80 elderly patients with lung cancer (aged > 60 years old) in the hospital from January 2019 to September 2020 were selected as the research objects. In addition, all research objects were diagnosed as lung cancer using percutaneous lung puncture, bronchoscopy biopsy, lymph node biopsy, and other biopsies of metastatic lesions. They were randomly divided into two groups, patients treated with pemetrexed combined with cisplatin were included in the pemetrexed group (40 cases aged 62∼83 years old) and patients treated with docetaxel combined with cisplatin were included in the docetaxel group (40 cases aged 61∼85 years old). There was no significant difference in age and gender between the two groups of patients (*P* > 0.05), and they were comparable (Table 1). This study had been approved by the ethics committee of hospital, and the patients and their families understand the situation of the study and sign the informed consent forms.

Inclusion criteria are as follows: patients with the systemic function status score (PS score) standard of below 2 points; patients whose estimated survival time was more than 3 months; patients whose liver and kidney function and blood routine before chemotherapy were in the normal range; and patients without severe qualitative lesions in important organs.

Exclusion criteria are as follows: patients who were ≤60 years old; patients with unclear clinical stage; patients with less than 2 cycles of chemotherapy; patients who did not cooperate; and patients with malignant tumors of other parts.

### 2.2. Treatment Schemes

Pemetrexed group: 40 patients with lung cancer started to take folic acid tablets 7 days before receiving pemetrexed chemotherapy (400 *μ*g/d) for continuous 21 days. They were given VitB12 (1000 *μ*g/time) intramuscular injection at the same time 7 days before chemotherapy, once every 21 days. Dexamethasone (4 mg) was taken orally 1 day before chemotherapy and 1st day and 2nd day after chemotherapy to prevent skin rash and allergies. Pemetrexed (500 mg/m^2^) was given in the form of intravenous infusion on the first day; cisplatin (70 mg/m^2^), intravenous infusion, was given on the first time. One cycle included 21 days.

Docetaxel group: the patients were given dexamethasone (5 mg) intravenously before chemotherapy, and docetaxel (75 mg/m^2^) and cisplatin (70 mg/m^2^) were intravenously injected on the first day, with 21 days for a cycle. Measures to prevent vomiting were given during chemotherapy. The blood picture was rechecked once a week, tumor markers and biochemical indicators were rechecked before each chemotherapy, and CT imaging was performed to evaluate the curative effect after 2 cycles of chemotherapy.

### 2.3. Evaluation Criteria

The curative effect was evaluated according to the solid tumor curative effect evaluation standard RECIST 1.1 given by World Health Organization (WHO) [13]. In this study, CT scans were used to evaluate the changes in the size of the measurable nodules before and after treatment. The imaging examination before treatment showed that there were 1 or more measurable lesions. All patients had at least 2 cycles of chemotherapy. The curative effect was evaluated after 2 cycles of chemotherapy and compared with the lesion before treatment. It should detect the serum tumor markers (carcinoembryonic antigen (CEA), cytokeratin fragment antigen 21–1 (CYFRA 21–1), and squamous cell-associated antigen (SCC)). In addition, the chest and abdomen were performed with the enhanced CT, and then it should continue to the next round according to RECIST standards treatment.

Evaluation indicators of target lesions included complete remission (CR), partial remission (PR), stable disease (SD), and disease progression (PD).

The response rate (RR) (RR = CR + PR), disease control rate (DCR) (DCR = CR + PR + SD), time to progression (TTP), median survival time (MST), and progression-free survival (PFS) were detected. The follow-up was performed using a combination of outpatient and telephone follow-up. During the follow-up, the survival time (months) of all patients was recorded.

### 2.4. CT Scan

The patient was in a supine position with the head advanced, and a 16-slice spiral CT scanner was used. Before the examination, the patient was trained to breathe and hold his breath calmly to eliminate tension. Local plain scan was performed on the mass and determine the largest level of the lesion on the plain scan image. Scanning conditions were set as follows: 120 kV, 200 mA, 1.0 s/r, acquisition layer thickness of 1 mm × 16, reconstruction interval of 7 mm, reconstruction layer thickness of 7 mm, and pitch of 15. Dynamic scanning was performed on selected layers according to cross-sectional positioning. Perfusion scanning conditions were set as follows: 120 kV, 200 mA, 1.0 s/r, field of view (FOV) of 40, matrix of 512 × 512, acquisition layer thickness of 2 mm × 4, and interval of 1 s. 50 mL of 300 mg/mL iohexo was injected intravenously in front of the elbow, 21 G × 3/4 intravenous indwelling needle was embedded through median cubital vein puncture, with the rate of 4 mL/s, delay of 6 s, and data acquisition of 32 s, generating 128 layers of perfusion images. After the completion of the perfusion scan, 50 mL of contrast medium was injected again at a rate of 2 mL/s, with a delay of 25 seconds for routine enhanced scan.

### 2.5. Nursing Methods

Psychological nursing should be implemented for all patients. Nursing staff should understand and support patients, meet their reasonable needs, fully mobilize the enthusiasm of patients' spouses or family members, assist patients with treatment with a good attitude, and respond to various adverse reactions in a timely manner.

Before administration, patients and family members should be informed that chemotherapy drugs may cause side effects such as leukopenia and thrombocytopenia. The importance of vitamin B12 and folic acid should be explained, the compliance of patients with self-medication outside the hospital has to be improved, patients should be encouraged to drink more water, urine output should be maintained above 2000 mL, and the color and nature of urine should be paid attention to.

Patients have different degrees of skin rash and itching, and the head, face, and trunk are more common. Oral antihistamines or hormone antiallergic drugs can be used for nursing, and antibiotics can be taken orally in a short time to prevent infection. Patients are advised to wear soft and breathable clothes, keep their skin clean, and if skin rash or itching occurs, they should avoid scratching and rubbing irritating drugs locally. Hydrocortisone or dexamethasone ointment can be applied for external treatment.

According to the nursing of gastrointestinal reaction, antiemetic drugs such as granisetron and metoclopramide should be used on time and correctly before treatment to prevent the occurrence of nausea and vomiting. It is important to reduce bad stimulation, create a good living environment, and keep the indoor air fresh and the temperature appropriate. Dietary guidance should be given. For patients with diarrhea, the number and characteristics of defecation should be recorded. Compound phenethylpiperidine can be taken orally to reduce gastrointestinal peristalsis. For patients with dehydration symptoms, water and electrolytes should be added intravenously.

Cisplatin has obvious toxic and side effects on kidney, and active hydration and diuresis during chemotherapy are the keys to prevent kidney damage. On the day of application of cisplatin, the hydration volume should be >2000 mL and furosemide 20 mg or 20% mannitol 125 mL should be given for diuresis. In order to ensure continuous renal perfusion and maintain the infusion time for more than 14 hours, patients should be encouraged to drink more water and keep sufficient urine output and the 24-hour urine output should be >2000 mL.

### 2.6. CT Image Based on DenseNet

**Figure 1:**
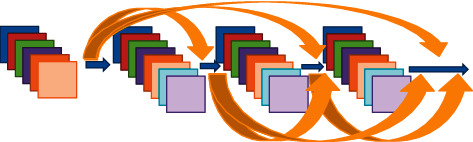
The dense connection mechanism of the DenseNet.

DenseNet is a dense connection mechanism that connects all layers to each other. That is, the input of each layer in the network is the output of all the previous layers [14].  Figure 1 showed the dense connection mechanism of the DenseNet.

If there were *N* layers in the DenseNet, there were *N*(*N* − 1)/2 connections in the entire DenseNet CNN. DenseNet is not only densely structured but also can improve network efficiency by connecting feature maps of different layers.

The output of the traditional convolutional neural network (CNN) in the N layer was given as follows:

(1) $$\begin{array}{c} x_{n}=T_{n}\left(x_{n-1}\right). \end{array}$$

In (1), *x* represented output and *T*_*n*_ represented a nonlinear transformation function, which was a series of comprehensive operations such as batch normalization (BN), ReLU activation function, pooling operation, and Conv convolution operation. In the DenseNet, the identity function from the input of the previous layer was added:

(2) $$\begin{array}{c} x_{n}=T_{n}\left(x_{n-1}\right)+x_{n-1}. \end{array}$$

At the same time, all the previous layers were taken as input:

(3) $$\begin{array}{c} x_{n}=T_{n}\left(\left[x_{0},x_{1},\ldots ,x_{n-1}\right]\right). \end{array}$$

Parameters of DenseNet were shown in Table 2.

### 2.7. Image Analysis and Processing

The two-dimensional (2D) images of the CT image sequence of 80 patients with nodules were extracted. The CT image was segmented, the candidate position was taken as the center to obtain the image block, and the cross-section, sagittal plane, and coronal plane were extracted. Due to the size of most nodules, the receptive field size of each image block was set to 64 × 64. The CT values were cut to (−1000–400 HU) and normalized to (0, 1). The average gray value was subtracted to fit the DenseNet.

### 2.8. Setting for Algorithm Comparison

The ratio of the training set to the test set was 8 : 2, and these two sets of pictures were used as the input of the DenseNet. When the model was trained, the network growth speed *k* was set to 32, the compression rate of the transition layer between different blocks was set to 0.5, the learning rate was set to 0.01, and the dropout rate was set to 0.3. The optimizer used the batch gradient descent method. In order to make the conclusion more convincing, the DenseNet network was compared with Let Net-5 and ResNet models in this study.

### 2.9. Statistical Methods

SPSS (Statistical Product and Service Solutions) 22.0 was adopted as the analysis and statistical software for statistical analysis. The count data were expressed in percentage (%),the data conforming to the normal distribution was expressed in $\bar{x}\pm s$, and the *t*-test was used to verify. The results were statistically significant when *P* < 0.05.

## 3. Results

### 3.1. Results of Image Segmentation Algorithms

**Figure 2:**
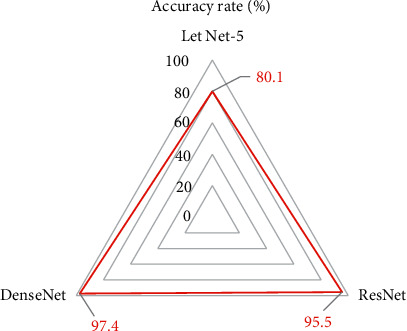
Comparison of the diagnostic test results of lung malignant nodules.

There were 156 malignant nodules in 80 elderly lung cancer patients. The DenseNet network was compared with Let Net-5 and ResNet models to diagnose malignant nodules. The results showed that the Let Net-5 network detected 125 malignant nodules, the ResNet network detected 149 malignant nodules, and the DenseNet network detected 152 malignant nodules. The diagnosis accuracy of the DenseNet network (97.4%) was higher than that of the Let Net-5 network (80.1%) and ResNet model (95.5%), and there was no statistical difference (*P* > 0.05), as shown in Figure 2. The diagnostic accuracy of DenseNet was better than other typical models, which proved the effectiveness of DenseNet in diagnosing malignant nodules.

**Figure 3:**
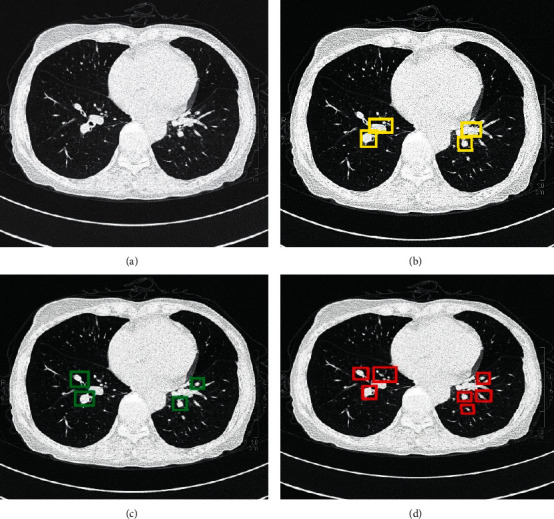
Comparison of CT image segmentation results. (a) Original CT image; (b) Image segmented by Let Net-5 network; (c) Image segmented by ResNet; (d) Image segmented by the DenseNet.

The CT image was segmented based on the DenseNet, and the results were shown in Figure 3. It was found that the DenseNet showed a more accurate and higher definition for the number of nodules diagnosed.

### 3.2. Clinical Efficacy and Survival

**Figure 4:**
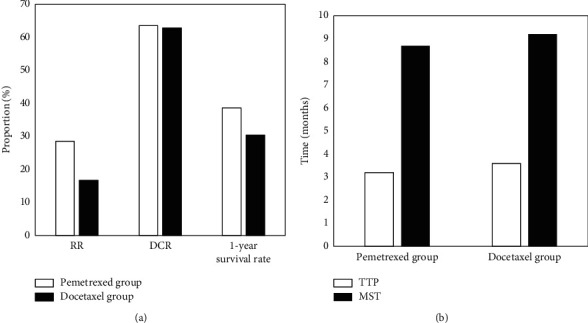
Comparison of clinical efficacy between two groups. (a) Comparison of clinical efficacy; (b) Comparison of survival.

The RRs of the pemetrexed group and the docetaxel group were 28.5% and 16.78%, respectively; the DCRs were 63.5% and 62.8%, respectively; and the 1-year survival rates were 38.6% and 30.4%, respectively, showing statistically obvious differences. The follow-up time for all patients was 6–24 months. Among them, the TTP of the pemetrexed group was 3.2 months and the MST was 8.7 months; the TTP of the docetaxel group was 3.6 months, and the MST was 9.2 months. There was no statistically significant difference in TTP and MST between the two groups of patients, as shown in Figure 4.

### 3.3. CT Imaging Changes of Lung Cancer before and after Chemotherapy

**Figure 5:**
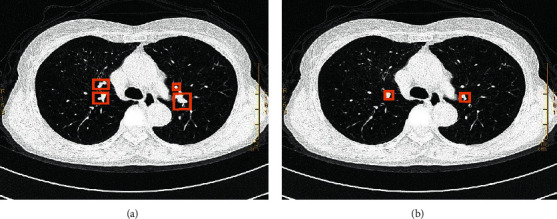
Changes in lung cancer CT images before and after chemotherapy. Male, 79 years old, patient with lung cancer. (a) Before chemotherapy, the CT image showed the distribution of nodular soft tissue density masses at the posterior and lower right hilum (the size was about 0.8 cm in diameter, the edges were slightly under-rectified, and there was no obvious swollen lymphoma in the mediastinum). (b) After chemotherapy, CT images showed that the posterior and lower right hilum nodules were significantly smaller than before treatment, with a diameter of about 0.4 cm, blurred edges, reduced right lung volume, and no obvious lymphadenopathy in the mediastinum.

The imaging features of lung cancer showed an irregular flaky consolidation at the tip and posterior segment of the lung, and the lesion was shrunken; dilated bronchioles were seen inside, with clear edges and long burrs; bilateral thoracic cavity, horizontal fissure, and bilateral oblique fissure showed fluid density shadow; left lower lung tissue was compressed, and middle and lower lobe scattered in the parenchymal zone in the right lung. In addition, the left atrium and left ventricle were enlarged, and multiple lymph nodes in the mediastinum were enlarged, as shown in Figure 5(a). CT was rechecked after chemotherapy. The lung cancer patients in the remission group had shrunken masses, unclear borders, and open atelectasis, as shown in Figure 5(b).

### 3.4. Determination of Tumor Markers

**Figure 6:**
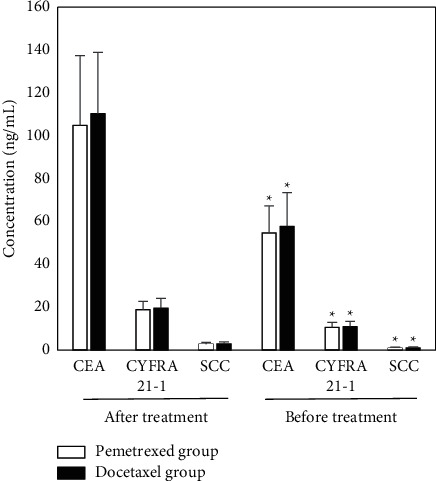
Tumor marker levels before and after treatment. ^*∗*^Compared with the levels before treatment, *P* < 0.05.

CEA, CYFRA21-1, and SCC after chemotherapy in the pemetrexed group and docetaxel group were lower than before chemotherapy, and the differences were statistically significant (*P* < 0.05). In addition, there was no significant difference in CEA, CYFRA21-1, and SCC before and after chemotherapy in the pemetrexed group and docetaxel group (*P* > 0.05), as illustrated in Figure 6.

### 3.5. Nursing Satisfaction

**Figure 7:**
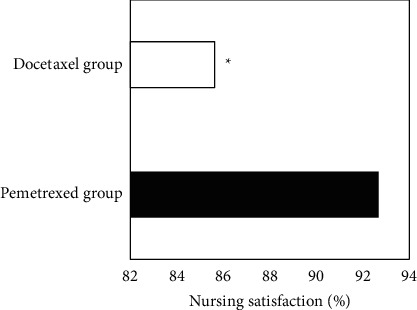
Comparison of nursing satisfaction. ^*∗*^Compared with the pemetrexed group, *P* < 0.05.

The satisfaction degree of nursing care in the pemetrexed group (92.67%) was significantly higher than that in the docetaxel group (85.62%), and the difference was statistically significant (*P* < 0.05), as shown in Figure 7.

### 3.6. Adverse Reactions

**Figure 8:**
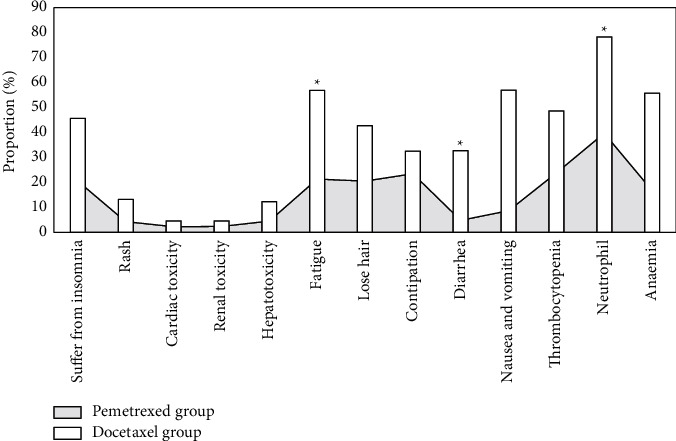
Comparison of adverse reactions between the two groups. ^*∗*^Compared with the pemetrexed group, *P* < 0.05.

The main adverse reactions of the two groups of patients were insomnia, skin rash, cardiotoxicity, nephrotoxicity, liver toxicity, fatigue, hair loss, constipation, diarrhea, nausea and vomiting, thrombocytopenia, neutrophils, anemia, etc. Adverse reactions such as fatigue, diarrhea, and neutrophils in the pemetrexed group were lower than those in the docetaxel group, and the difference was statistically significant (*P* < 0.05), while the difference in other adverse reactions was not statistically obvious (*P* > 0.05). The adverse reactions were shown in Figure 8.

## 4. Discussion

Early diagnosis of lung cancer is very difficult. Most patients are already in the middle and late stages when they are diagnosed, especially elderly patients. They even have distant metastases and miss the chance of radical surgery. With the aging of the population intensifying, the incidence of lung cancer among the elderly is increasing [15]. Due to the weakening of the physiological functions of the elderly, liver reserves, renal clearance, and lung function are greatly reduced, and hematopoietic function is attenuated. If other organ diseases are combined at the same time, the chemotherapy tolerability of elderly lung cancer patients will decrease. The continuous updating of chemotherapy drugs in recent years has brought good clinical effects for the treatment of elderly lung cancer patients [16]. Therefore, this study used a comprehensive approach of chemotherapy combined with radiotherapy for patients with advanced NSCLC. The platinum-containing dual-drug combination chemotherapy regimen for the treatment of advanced NSCLC had been affirmed by a number of studies at home and abroad, and its clinical efficacy and survival rate had been greatly improved. This study retrospectively analyzed the changes of CEA, CYFRA 21–1, and SCC tumor markers, and explored the clinical efficacy of pemetrexed combined with platinum on NSCLC chemotherapy.

Pemetrexed shows the characteristics of low toxicity, high efficiency, and extensive antitumor properties. It was approved by the Food and Drug Administration of the United States in 2004 for the second-line treatment of advanced NSCLC. Previous clinical studies have shown that pemetrexed used in the second-line treatment of lung cancer exhibits a good remission rate and overall survival advantage. Gadgeel [17] found that pemetrexed and docetaxel have different therapeutic effects for different histological types, while pemetrexed treats NSCLC patients with PFS longer. Cisplatin is a nonspecific antitumor drug targeting the cell cycle and is currently widely used in clinical practice. Cisplatin is still active on anaerobic cells and aerobic cells, so it has a radiosensitization effect [10, 18, 19].

CEA is the most commonly used tumor marker in clinical practice, and its level is related to tumor metastasis and recurrence. Studies have shown that about 20% of lung cancer patients have higher CEA levels, which means that dynamic monitoring of CEA levels can reflect the clinical efficacy of lung cancer [20]. CYFRA21-1 is a fragment of cytokeratin 19, which is very common in patients with NSCLC. The RR of advanced NSCLC imaging assessment of chemotherapy is positively correlated with the assessment of CEA and CYFRA21-1. SCC is not elevated in small cell lung cancer, but it is abnormally elevated in lung squamous cell carcinoma. The detection of SCC can help differentiate NSCLC [21]. CEA, CYFRA21-1, and SCC in the pemetrexed group and docetaxel group after chemotherapy were lower than those before chemotherapy (*P* < 0.05). It shows that pemetrexed combined with cisplatin has a good effect in the treatment of NSCLC, which is consistent with the research results of Chen et al. (2020) [22]. Pemetrexed inhibits the activities of the above three key enzymes through multiple targets, reduces the synthesis of purines and pyrimidines, and ultimately inhibits the production of tumor cell DNA. In particular, tumor cells stagnate during the DNA synthesis stage of mitosis, leading to tumor cell apoptosis and achieving the goal of antitumor.

The folic acid and vitamin B12 were given accurately before chemotherapy to reduce bone marrow suppression and accurately take dexamethasone to reduce the occurrence of skin toxicity. Medical staff should closely observe the toxic and side effects of drugs, deal with them promptly if they find undesirable conditions, strengthen patients' psychological care and health knowledge education, and obtain the support and cooperation of patients' families. Adverse reactions such as fatigue, diarrhea, and neutrophils in the pemetrexed group were lower than those in the docetaxel group, and the differences were statistically obvious (*P* < 0.05). The results showed that there were no serious adverse reactions in the two groups of patients after treatment, and the adverse reactions of pemetrexed combined with cisplatin were mild.

## 5. Conclusion

In this study, 80 patients with lung cancer were selected as the research objects, and the clinical efficacy of pemetrexed combined with platinum and docetaxel chemotherapy was compared under the guidance of CT images based on DenseNet. DenseNet convolutional neural network has high diagnostic accuracy; methotrexate combined with platinum chemotherapy can improve the chemotherapy effect in elderly patients with lung cancer, with a low degree of adverse reactions and good overall tolerance, which can be used as the first-line treatment for elderly patients with lung cancer. The shortcoming of this study was that the number of included cases was small and data bias could not be ruled out. In the later stage, further research was needed to explore the differences in the efficacy and adverse reactions of the two programs and to provide a basis for individualized treatment of patients. In conclusion, it was confirmed in this study that pemetrexed combined with cisplatin showed good clinical efficacy in the treatment of elderly lung cancer and was worthy of promotion.

## Figures and Tables

**Table 1:** Clinical characteristics of the two groups of patients before treatment (*n*, %).

		Pemetrexed group (*n* = 40)	Docetaxel group (*n* = 40)	*P* value
Average age (years old)		73.2 ± 12.5	74.5 ± 13.8	>0.05

Gender	Males	26 (65%)	24 (60%)	>0.05
	Females	14 (35%)	16 (40%)	>0.05

PS score	0	6 (15%)	9 (22.5%)	>0.05
	1	33 (82.5%)	24 (60%)	>0.05
	2	1 (2.5%)	7 (17.5%)	>0.05

**Table 2:** Parameters of DenseNet.

Layer	Output size	Parameter
Convolution	112 × 112	7 × 7 conv, stide2
Pooling	56 × 56	3 × 3 max pool, stide2
Dense block	56 × 56	$\left(\begin{array}{c} 1\times 1conv\\ 3\times 3conv \end{array}\right)$ × 6
Transition layer	56 × 56	1 × 1 conv
	28 × 28	2 × 2 average pool, stide2
Dense block	28 × 28	$\left(\begin{array}{c} 1\times 1\text{conv}\\ 3\times 3\text{conv} \end{array}\right)$ × 12
Classification layer	1 × 1	7 × 7 global average pool

## Data Availability

The data used to support the findings of this study are available from the corresponding author upon request.
